# Evidence for Chemical Vapor Induced 2H to 1T Phase Transition in MoX_2_ (X = Se, S) Transition Metal Dichalcogenide Films

**DOI:** 10.1038/s41598-017-04224-4

**Published:** 2017-06-19

**Authors:** Adam L. Friedman, Aubrey T. Hanbicki, F. Keith Perkins, Glenn G. Jernigan, James C. Culbertson, Paul M. Campbell

**Affiliations:** 1 Materials Science and Technology Division, Naval Research Laboratory, 4555 Overlook Ave., S.W., Washington, DC 20375 USA; 2 Electronics Science and Technology Division, Naval Research Laboratory, 4555 Overlook Ave., S.W., Washington, DC 20375 USA

## Abstract

Electron-donors can impart charge to the surface of transition metal dichalcogenide (TMD) films while interacting with the film via a weak physisorption bond, making them ideal for vapor and gas sensors. We expose monolayer MoS_2_ and MoSe_2_ films to strong electron-donor chemical vapor analytes. After analyzing the resultant behavior and taking into consideration doping effects, we conclude that exposure to strong electron-donors could be a method of inducing the semiconductor-metal 2H-1T TMD phase transition. We find that the conductance response to strong electron donors in both monolayer MoS_2_ and MoSe_2_ FET devices ceases after moderate exposure, with final value of the conductance being on order of that expected for the 1T phase. Full device relaxation back to a semiconducting state is accomplished by annealing in vacuum at 400 °C. We also examine chemically exposed TMD films intermittently interrogated with Raman and photoluminescence spectroscopy. We observe the appearance of weak characteristic 1T phase Raman features for MoS_2_ and we observed a quenching of the photoluminescence of both TMD films that is recoverable with annealing. Considering all of our data together, the effects cannot be described by doping alone. Additionally, our results suggest a mechanism for a new type of passive chemical vapor sensor.

## Introduction

Since the discovery in 2004–2005^1^ that single monolayer films of transition metal dichalcogenides (TMDs) can be isolated from the bulk due to weak interlayer van der Waals bonding, these materials have continued to reveal new and remarkable behaviors and properties. Monolayer TMDs offer possible advances in technology over current material paradigms, paving the way for inexpensive, flexible, high-performance devices that exploit their unique surface-dominated functionality. Abbreviated chemically as MX_2_, where M is a transition metal (Mo, W, Nb, Hf, Ta, V, etc.) and X is a chalocogen (S, Se, or Te) the monolayer TMDs include insulators, semiconductors, metals, and other types of materials with a variety of properties not observed in the bulk. For example, the materials MoX_2_ and WX_2_, are semiconductors that transition from indirect gap in the bulk to direct gap^2, 3^ as monolayers and have shown field effect transistor (FET) on/off ratios and room temperature mobilities that are competitive with the state-of-the-art^4^. Additionally, these materials are extremely promising for chemical vapor sensing applications because the inherent few-atom-thickness of the material greatly enhances their sensitivity to even the smallest surface perturbations. Certain films respond selectively to strong electron donors through a physisorption process^5, 6^. A minute quantity of analyte lying on the surface of the TMD acts as an electron donor and local reducing agent, which measurably affects the conductance of the film^5–7^.

**Figure 1:**
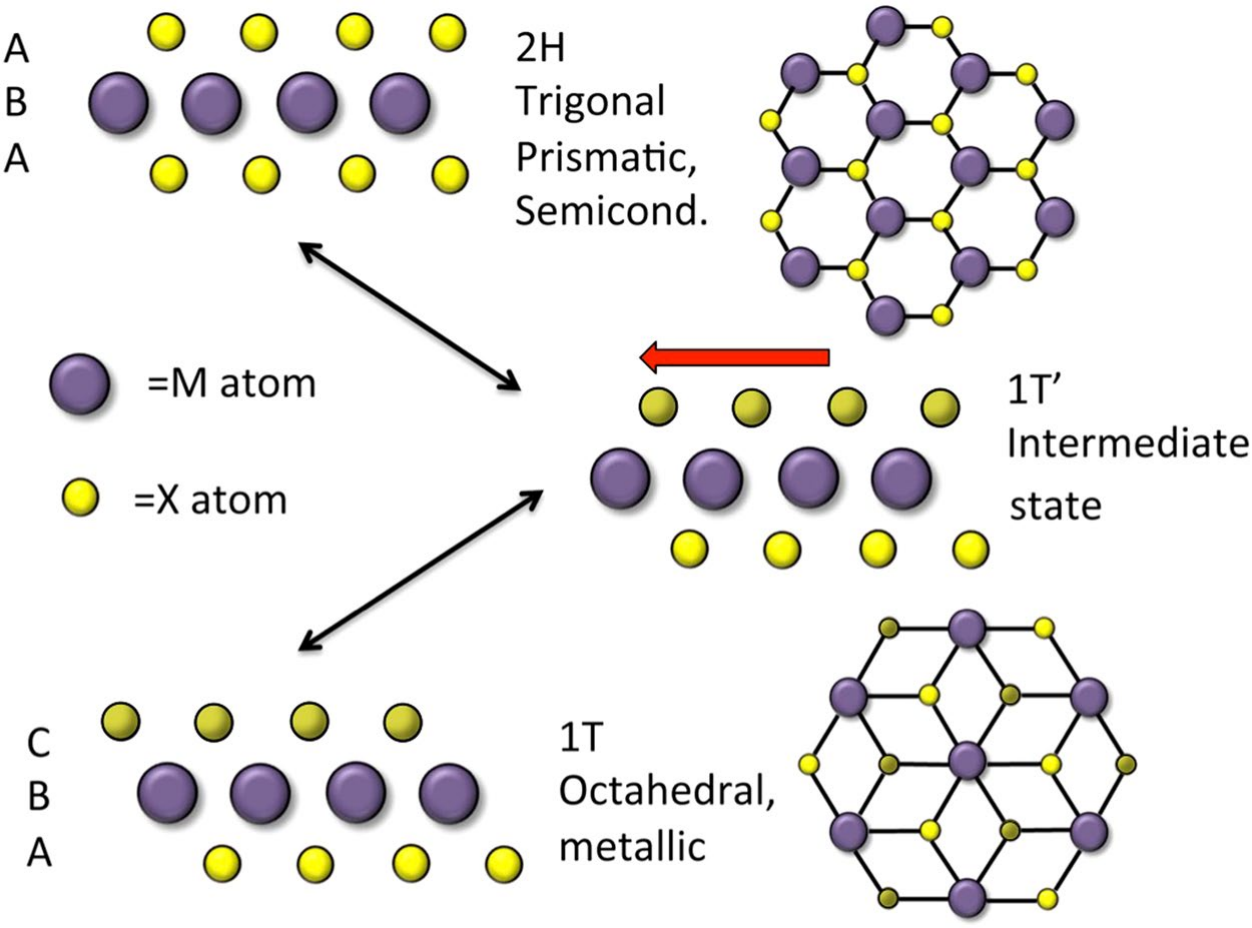
Model of the 2H-1T phase transition in MoX_2_ films. The 2H phase is trigonal prismatic and the material is a semiconductor. The 1T phase is octahedral and the material is metallic. The 1T’ phase is an intermediate state.

Recent interest has intensively focused on the phase change properties of MX_2_ films. The semiconductor-metal 2H-1T phase transition in TMDs creates a route towards FET-based electronic device engineering of either the channel or the contacts in a reversible, repeatable, and non-damaging yet robust way^8, 9^. The 2H-1T transition is driven by excess strain or charge in the TMD lattice. For most of the TMDs, the lowest energy, stable configuration is the semiconducting, tetrahedral 2H formation, where the three chemical planes in a single monolayer are stacked in an A-B-A sequence. Lattice strain in the TMD, which can be caused by excess charge delivered by surface dopants, can force the material first into an intermediate, unstable 1T’ state and then into the metastable metallic, octahedral 1T state. Here, the atomic planes are stacked as C-B-A in a local energy minimum. Schematic depictions of these various phases are presented in Fig. 1. Each TMD requires a different amount of energy to transition between phases^10^. For the MoX_2_ films, which will be the focus of this letter, the energies required for a 2H to 1T transition ΔE(2H→1T) are such that ΔE[MoS_2_] > ΔE[MoSe_2_] > ΔE[MoTe_2_]. Excess charge from adsorbates either on or otherwise incorporated in the TMD can also stabilize the 1T state, which can be relaxed back into the 2H state by thermal annealing. This was demonstrated experimentally by treating a MoS_2_ monolayer with *n*-butyllithium, a strong electron donor^11, 12^. Even after the *n*-butyllithium was completely removed from the surface and was no longer actively donating charge to the film, the film remained in the 1T phase.

It can be challenging to differentiate experimentally between the 2H and 1T phases and further differentiate a phase transition from a large increase in doping, although a variety of methods are widely accepted in the literature. Conductance measurements provide perhaps the most unequivocal means of discriminating between the 2H and 1T phases, with the metallic phase offering a conductance that is significantly higher than the semiconducting phase^8^. Although a highly-surface-doped 2H phase film can be very conductive^13^, only a 1T-phase film will show an abrupt change in conductance at the transition point and remain relatively stable in a variety of conditions (vacuum, annealing below the transition temperature, etc.) that would otherwise remove surface dopants and cause a loss of conductance for the surface doped 2H-phase film. Additionally, detailed microscopy measurements such as transmission electron microscopy (TEM) can provide a direct method for visualizing the different phases. However, because these two phases have similar lattice constants and symmetries, and since monolayer samples have small TEM imaging cross sections, such measurements can be extremely challenging^11^. Moreover, due to transferability and optical contrast considerations, the most commonly studied high-quality mechanically exfoliated films are not easily amenable to TEM studies. Thirdly, optical measurements can be used as differentiation methods because the photoluminescence (PL) readily observed from the semiconducting phase is quenched in the metallic phase^14^. However, because multiple phases can exist in the same TMD film, one cannot expect a complete suppression of PL for a partially transitioned film, but at most a partial reduction in PL^15^. Additionally, doping effects can also cause a reduction in PL, so other methods must be used concurrently for accurate phase identification^7, 16^. The TMD MoS_2_, when very thin, has also been shown to exhibit additional Raman features (identified as J1, J2, and J3) when it transitions from 2H to 1T, but these peaks can be very weak and difficult to discern if both phases coexist^14^. Moreover, recent studies show that most common methods of inducing a 2H to 1T transition leave as much as 20–50% of the film in the 2H phase, resulting in a variety of possible discrepancies in the literature as to the Raman behavior of a true 1T phase film^17^. Finally, XPS measurements have indicated a shift of ~1 eV in binding energy for the Mo 3d_3/2_ and 3d_5/2_ peaks in the 1T phase as compared to 2H phase and a corresponding shift in the S 2p_1/2_ and 2p_3/2_ peaks^15^. However, these peak shifts are identical for doped 2H films and the S peak shifts can alternatively be caused by vacancies or defects^13, 18^. Overall, optical interrogation and XPS methods cannot definitively prove that a phase transition has occurred, but can corroborate other results and support each other.

In this study, we demonstrate evidence for a chemical vapor induced 2H-1T phase change in monolayer MoSe_2_ and MoS_2_ films. We actively monitored the conductance of TMD devices as a function of exposure to various analytes. In an ambient atmosphere intermittently containing dilute vapors of strong electron-donor analytes, there was both a significant increase in conductance as well as an attenuation of chemiresistance response after an observed cumulative exposure. The higher conductance state persisted even after all analyte had almost certainly desorbed from the film surface, and the samples recovered their original optical and electronic properties after annealing above the likely transition temperature. These behaviors support the conclusion of a vapor-induced partial phase change. Finally, to provide further corroborating evidence that the MoX_2_ films undergo the phase transition, we used Raman and photoluminescence spectroscopy (PL) to characterize the films before and after exposure to strong electron donor analytes.

## Experiment and Analysis

Films of MoSe_2_ and MoS_2_ were mechanically exfoliated from bulk crystals and deposited on 275 nm thermally grown SiO_2_ on n^+^ Si substrates. Monolayer films were identified using optical contrast in a metallurgical compound microscope and confirmed using micro-spot (~1 µm) PL and Raman spectroscopy. The PL and the Raman spectroscopies were performed using 532 nm and 488 nm excitations, respectively, both in ambient conditions^19, 20^. We used less than 50 µW power with the 532 laser and less than 10 µW from the 488 laser to prevent damage to the films caused by local heating or other spurious effects^21^. Using Raman spectroscopy, the MoS_2_ monolayer thickness was confirmed by measuring a separation of 18.1 cm^−1^ between the A_1g_ and E^1^_2g_ mode peaks^2, 22^. The MoSe_2_ monolayer was identified by an inactive B_2g_ mode (expected at ~353 cm^−1^) that is observable in few-layer films due to a loss of translation symmetry but absent in monolayers^23^. Monolayer thickness was further confirmed by observation of the strong PL peak due to the A-exciton emission characteristic of the direct bandgap at monolayer thickness for both TMD films^19, 20, 22^. Emission significantly decreases for the indirect gap bilayer film, and then almost completely vanishes for even thicker films. Experiments were not performed on MoTe_2_, because without a capping layer, it quickly oxidizes in atmosphere, rendering it unfeasible for real-world chemical vapor sensing applications^24^.

**Figure 2:**
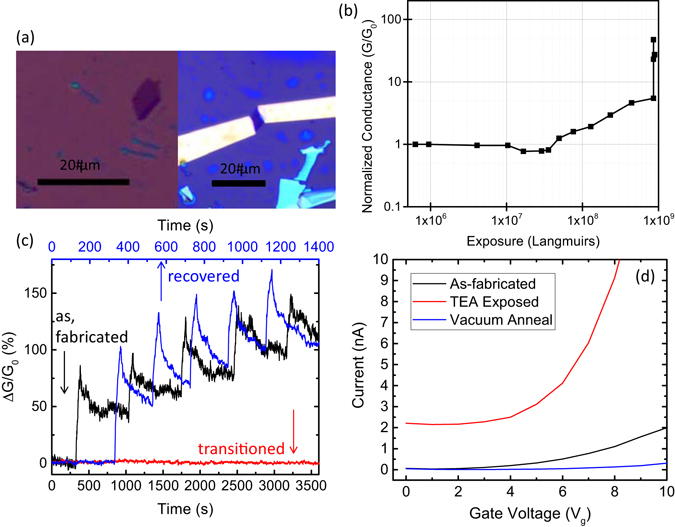
(**a**) Optical image of a monolayer MoSe_2_ flake (left) and a MoSe_2_ FET device fabricated from that flake (right). (**b**) Normalized MoSe_2_ device conductance as a function of chemical vapor exposure for a typical device. The device stopped responding to analyte vapor at the point where the conductivity is observed to abruptly rise, about 8 × 10^8^ Langmuirs. Annealing resulted in the recovery of the original conductance and sensor response. (**c**) Response to a pulsed sequence of 0.04% P_0_ BuAm vapor. The black line shows the sensor response of the as-fabricated device to a series of pulses (60 s on, 645 s off), and the red line shows an unresponsive sensor corresponding to 9 × 10^8^ Langmuirs in (**b**) and with the same series of pulses. Both of these traces go with the bottom x-axis. The blue line (goes with the top x-axis) shows the recovered response of the device to a series of pulses (30 s on, 160 s off) after annealing at 400 °C in vacuum for 2 hours. (**d**) Current vs. back gate voltage taken at 1 V for before exposure, after exposure, and after annealing the device.

Chemical vapor exposure was performed using two different methods for either active or passive exposure monitoring. Active sample monitoring was accomplished electrically in a home-built chemical vapor sensing apparatus, described previously^5^. Devices were fabricated with Ti/Au (5 nm/ 35 nm) electrical contacts defined using electron-beam lithography in PMMA followed by electron-beam evaporation and lift-off in acetone. The n^+^ Si substrate was used to provide a back gate electrode. Further details of the device fabrication process and basic FET behavior are found elsewhere^5, 6, 9^. Optical images of a monolayer MoSe_2_ film before and after processing into a completed device are shown in Fig. 2a. Devices were then contacted with probes attached to computer-controlled lock-in amplifiers, either a low-impedance voltage source V_s_ = 0 V_dc_ + 0.1 V_ac,rms_ with frequency on the order of 2 kHz, or a high impedance (1 MΩ) bias resistor R_b_ in parallel with the 10 MΩ input impedance of a lock-in amplifier, where the lock-in amplifier measures the frequency-matched voltage drop across the resistor. In this way, we could independently measure small voltage changes ΔV across R_b_ corresponding to small changes in differential conductivity (G − G_0_)/G_0_. The devices were placed on a sample chuck with heating capabilities for *in situ* device annealing, with the temperature being monitored by a thermocouple contacting the top face of the substrate. Heating the sample during measurements enabled the devices to recover more quickly after chemical vapor dosing^9, 25^. Devices were constantly under ac source-drain bias and 20 V_dc_ gate-source bias while the films were intermittently exposed to vapors of butylamine (BuAm) or triethylamine (TEA), both strong electron donors, diluted under computer control into a flowing (5 lpm) dry high purity N_2_ ambient. As previously stated, MoS_2_ sensor devices have high selectivity for strong electron donor compounds due to physisorption caused by charge transfer into the film^5, 6, 9^. As expected, given the similar electronic and crystalline structures, MoSe_2_ behaves similarly^26^.

Figure 2b shows the normalized conductance of a MoSe_2_ device taken during a series of intermittent analyte exposures performed over four days and measured in Langmuirs. Although this unit is more typically associated with studies performed under ultra-high vacuum (UHV) conditions, we find it convenient and not without precedent^27, 28^ if we assume that the N_2_ ambient does not interact chemically with the surface of the film. Throughout the experiment, pure N_2_ gas (except for the addition of dilute analyte) was flowing over the substrate, the substrate was heated to about 40 °C, and the device was illuminated by white light. Exposures to BuAm or TEA ranged between concentrations of 1.4 × 10^3^ to 2.8 × 10^5^ ppm, over durations between 30 and 300 seconds. Intervals between exposures varied from 300 seconds to 18 hours. We observed no significant change in conductivity until about 4 × 10^7^ Langmuirs integrated exposure, followed by an increase described well by a power law tending to saturation, until about 9 × 10^8^ Langmuirs and an abrupt and essentially terminal increase of approximately two orders of magnitude higher conductance. At this time, we cannot explain the dip in normalized conductance observed at ~2 × 10^7^ Langmuirs, but it could be due to stochastic effects. Theoretical calculations and TEM measurements have determined that local strain (here provided by charge donation) can produce mixed phase films^10, 11^. Therefore, it is likely that a phase change is occurring in the vicinity of the adsorbed analytes where charge transfer is locally transitioning the lattice with increasing coverage and doping.

For one device, we measured the as-fabricated device conductance to be ~1.4 μS. This value is typical of MoSe_2_ devices, especially those with Schottky contacts^29, 30^. At the end of the exposure experiments, we measured the device conductance to be ~500 μS. In general, the conductivity increase was approximately 1.5 to 2 orders of magnitude. This conductivity increase is consistent with a 2H-1T, phase transition^8^. Further exposure to BuAm or TEA did not lead to a further evolution of conductivity, nor did annealing for five days at 55 °C in flowing N_2_ under the measurement conditions described above, nor seven days at 28 °C on the shelf. Furthermore, the chemiresistive response of the devices recorded during exposure was observed to vanish after the above exposures, as shown in Fig. 2c. In this curve, the black line (bottom axis) shows the conductance response of the device to a series of pulsed 0.04% P_0_ BuAm exposures. After a certain exposure threshold, we observed no response, shown in the red line (bottom axis). This behavior is consistent with a transition from a semiconducting to a metallic state, where added charge from adsorbates would have little to no effect and a charged adsorbate would be quickly screened and neutralized before eventually desorbing in regions that transitioned to the metallic state. Although a highly doped MoSe_2_ device would display greatly increased conductivity and a smaller response to pulsed vapors, it would not show an abrupt and terminal change in conductivity and the complete cessation of response to pulsed analyte vapors. At this point, this device was annealed in vacuum. The device response did not recover until annealed at 400 °C for 2 hours, with lower temperature vacuum annealing failing to result in recovery. The initial conductance was essentially recovered by the 400 °C anneal, as well as a chemiresistive response (shown in Fig. 2c, blue line, top axis), indicating a likely transition back to the 2H phase.

A possible source of the observed decrease in resistance could be additional or continuing surface doping from the analyte. Indeed, previous research shows that significantly lower resistances and orders of magnitude higher carrier concentrations are found in MoS_2_ films contacted by surface-stabilized, amine-based polymer films formed by liquid chemistry methods, and hence continuously doped^16^. When the amine-based film is removed from the MoS_2_ by annealing, the initial properties recover. However, density functional theory (DFT) analysis of adsorption on MoS_2_ films predicts that most molecules only weakly physisorb to the surface of the film and will spontaneously desorb under ambient conditions^31, 32^. Exceptions include sulfur containing thiols and certain oxygen containing species, none of which we used in this study, that can both physisorb to the surface of the film and chemisorb at defect sites. Nonetheless, thermal desorption spectroscopy studies demonstrated that even these more deeply-bound molecules will spontaneously desorb well below room temperature^33^. More importantly, these studies concluded that volatile organic compounds and NH_3_, which are very similar to the analytes used here^34^, only very weakly physisorb to the surface with adsorption energies in the range of 10–200 meV, depending on details of the adsorption site chemistry and the DFT model used^31, 32^. This is on order of kT (~25 meV) for our sensing conditions. Thus, we conclude that simple persistent doping is unlikely because the analyte molecules are known to desorb from the film quickly under the conditions that we use for our experiments. Moreover, we observe no change in conductance or optical behavior (discussed below) after placing the sample in vacuum or vacuum annealing at temperatures below the likely transition temperature (~400 °C), after which it is even more unlikely that even the most strongly adsorbed dopant will remain adsorbed. We therefore conclude that it cannot be doping effects that are causing our observed behavior.

Figure 2d shows current vs. back gate voltage at a constant 1 V source-drain bias for a device before exposure, after terminal exposure, and after annealing in vacuum at 400 °C. If a device is increasingly doped until the film changes phase in its entirety, we would expect to see an upwards shift in conductance with each successive exposure until we finally get a flat line at high conductance, similar to previous studies^16^. However, a partial phase change creates a film that has metallic islands or ribbons imbedded in a semiconductor. Similar hybrid semiconductor-metal structures have been studied in the past and found to behave differently than the picture expected for increasing doping. For example, some experiments have demonstrated a shifting between multiple conduction mechanisms including tunneling, variable range hopping, and thermally activated regimes that are all affected by bias and electric field^35^. Such a mixed semiconductor-metal material FET can result in an anomalously large electroconductance, governed by the size of the metallic islands^36^. The metallic areas act as current shunts in zero electric field, with most of the current flowing through them. In an applied electric field, there are more current paths around the metallic regions as the resistivity of the semiconductor in this region is reduced. In other words, electrons will take the path of least resistance, which in the off state of the FET would be some form of percolative motion through the metallic islands. However, when the gate is turned on, drastically lowering the resistance of the surrounding semiconducting regions, parallel conductance through both metallic and semiconducting regions becomes much more likely. This would actually make it *easier* to gate modulate the device after the partial phase change, similar to what is shown in Fig. 2d. These data are in strong agreement with the partial phase transition hypothesis, but not in agreement with the doping hypothesis. Additional electrical characterization can be found in the Supplemental Information.

Pulsed analyte vapor response curves similar to that in Fig. 2c were observed in previous studies and were described with a model that included two recovery components after the analyte flow was switched off^9, 37^. In the first component, the weakest of the physisorbed molecules desorb, resulting in a rapid decrease in conductivity (the “fast recovery”). Then, the more strongly adsorbed molecules desorb over a longer time, resulting in a slower decrease in conductivity until the sample has recovered, noted by a flat conductance line (the “slow recovery”). The timescale of this total process is on order of hundreds of seconds. This type of behavior is not observed in TMD sensors exposed to stronger bonding thiols or oxygen species. With these types of dopants, there is no recovery or cessation of analyte responsive conductance modulation^37, 38^. We would expect that if the adsorption was mostly due to bonding at defect sites, a similar behavior would result, which is clearly not what we observe. This is most likely because once the chemisorption binding sites are saturated, these compounds can still actively physisorb/desorb at weaker binding sites. This is further evidence that doping by persistent adsorbed analytes is not the cause of our observed behavior.

As discussed above, further corroborating evidence of a chemically induced phase transition can be gleaned from passive optical measurements of analyte-exposed films. In these experiments, no electrical signals were applied, and the substrate was at room temperature (25 °C) and in darkness. Here, as-exfoliated MoX_2_ films are placed in a small (100 mL) sealed bell jar containing an open vial of tripropylamine (TPA), another strong electron donor. This analyte was chosen for these experiments because of its lower vapor pressure (P_0,TPA_ = 2.2 × 10^3^ ppm as compared to P_0,BuAm_ = 1.4 × 10^5^ ppm, P_0,TEA_ = 7.1 × 10^4^ ppm)^39^, thus making it possible to generate vapor and expose the film without a directed flow. Moreover, the analyte spontaneously and immediately desorbs when the film is removed from the bell jar, as discussed above, but at a rate commensurate with a relatively low vapor pressure. The films were periodically removed from the bell jar for PL and Raman measurements and were replaced in the bell jar for further vapor exposure.

**Figure 3:**
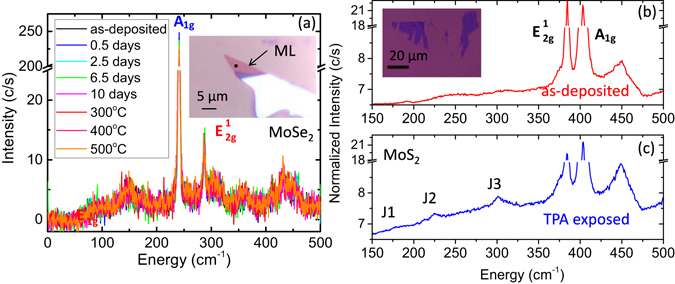
(**a**) Raman spectra taken on the monolayer MoSe_2_ film shown in the inset after a series of exposures to TPA in a bell jar and annealing steps, in order as indicated. The black dot on the optical image in the inset indicates where the spectra were measured. The relevant peaks are labeled. No significant changes are observed. (**b**) Raman spectra are taken on the monolayer MoS_2_ film shown in the optical image in the inset. This shows the spectrum from the as-deposited film. (**c**) The spectrum after 7 days exposure to TPA. The relevant peaks are labeled. Each line is an average of 10 points on the film. The appearance of the J1, J2, and J3 peaks in the exposed film is an indicator of the 1T phase.

Figure 3a shows Raman spectra from the monolayer MoSe_2_ film shown in the inset after a series of exposures to TPA for 0.5, 2.5, 6.5, and 10 days, corresponding respectively to cumulative exposures of 72, 360, 940, and 1400 × 10^9^ Langmuirs. The black dot in the inset image shows the position at which the presented spectra were measured. There are a variety of features in these spectra including sharp peaks at the A_1g_ (241 cm^−1^) and E^1^_2g_ (287 cm^−1^) energies, as well as a series of broad peaks from second order Raman processes which are not labeled. No obvious changes in the Raman spectrum were induced by analyte exposure. Secondary interactions or a breakdown of local symmetry, such as caused by a phase change, could possibly lead to the formation of new broad peaks in the spectrum. While it is still possible that there are additional peaks in our data, any new features are well within the noise of our measurement. To the best of our knowledge, there is no information in the literature on the Raman spectrum of 1T MoSe_2_. Further Raman analysis can be found in the Supplemental Information. After 10 days of TPA exposure, we annealed the sample in vacuum at 300 °C, 400 °C, and 500 °C for 2 hours each time, taking measurements between each annealing step. We again observed no measurable change compared to the initial state of the film.

Figure 3b,c shows Raman spectroscopy data from the monolayer MoS_2_ film shown in the inset. Figure 3b shows the Raman data from an as-deposited 2H phase film and Fig. 3c shows the Raman data collected after the film was exposed to TPA for 7 days (1 × 10^12^ Langmuirs). After exposure, the J1 (156 cm^−1^), J2 (226 cm^−1^), and J3 (300 cm^−1^) mode peaks were weakly visible, but still easily discernable^14, 40^. These peaks are expected for the 1T phase due to superlattice distortions but are absent in the 2H phase^14, 40^. In previous experimental studies where a 2H-1T phase change was initiated with an aqueous *n*-butyllithium treatment, the J1, J2 and J3 peaks were also observed with a similar width, but with greater relative intensity than reported here^14^. In a fully metallic phase film, these peaks are at slightly different positions and even greater in relative intensity^17^. However, in previous studies^14^ when the 2H and 1T phase coexisted in the same film, there were weak J1, J2, and J3 peaks, similar to our own measurements and suggesting that our data support a partial transition to the 1T phase, consistent with the previously discussed electrical measurement results. After annealing at 400 °C in vacuum for 2 hours, the film recovered to the 2H-like state.

**Figure 4:**
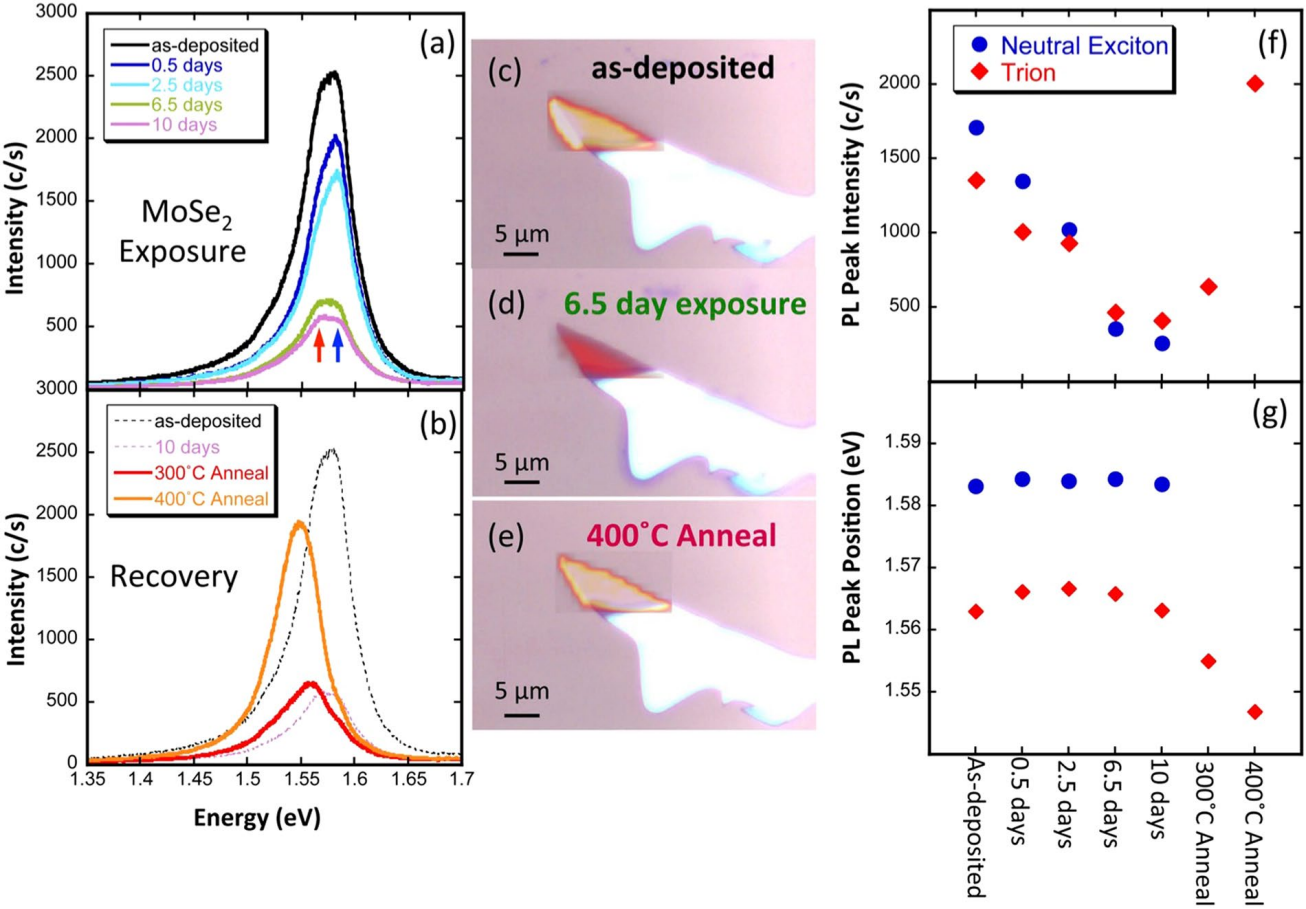
(**a**) PL spectra of the monolayer MoSe_2_ film shown in the inset of Fig. 3a after a series of exposures to TPA in a bell jar, as indicated. The red and blue arrows indicate the trion and neutral exciton, respectively. The as-deposited film lost ~80% of its intensity after 10 days of exposure, indicating a partial transition to the 1T phase. (**b**) PL spectra on the same MoSe_2_ film after a series of annealing steps compared to the as-deposited and partially transitioned film, as indicated. After annealing at 400 °C, the film recovered to within ~80% of the original intensity, indicating a transition back to the 2H phase. (**c**–**e**) PL peak intensity maps superimposed onto the optical image of the MoSe_2_ film showing both the decrease of intensity/recovering of the film and the uniformity of the emission. (**f**) PL peak intensity as a function of event further elucidating the effect of TPA exposure and annealing. (**g**) PL peak position of the two peaks indicated by the arrows in (**a**) as a function of exposure/annealing event. In (**f**) and (**g**), the blue circles are data for the neutral exciton, the red diamonds are for the trion, and error bars are on order of the symbol size.

Figure 4a shows PL spectra taken concurrently with the Raman data shown in Fig. 3a from the passively exposed MoSe_2_ film. In these spectra, there are clearly two components. We assign the higher energy component (blue arrow) to the neutral exciton and the lower energy component (red arrow) to the charged exciton, or trion^41^. Fig. 4b shows PL spectra on the same MoSe_2_ film after a series of vacuum annealing steps compared to the as-deposited and partially transitioned film, as indicated. It is evident that after a 400 °C vacuum anneal, the intensity has mostly recovered. In general, the oscillator strength of the trion is less than the neutral exciton for all MX_2_ films^21^. Therefore, we can conclude that the recovery is likely complete. Figure 4c–e show PL integrated intensity maps superimposed over the sample microscope image for **(c)** the as-deposited flake, **(d)** after 6.5 days exposure (940 × 10^9^ Langmuirs), and **(e)** after the 400 °C anneal, respectively.

The evolution of the intensity of the PL emission from the neutral exciton (blue circles) and trion (red diamonds) as a function of exposure are detailed in Fig. 4f along with the peak position as a function of event (either exposure or annealing) in Fig. 4g. In these figures, the plotted intensities and positions were derived from the spectra in Fig. 4a,b. As the sample is exposed to TPA, the intensity of both components decreases steadily with a general shift in spectral weight from the neutral exciton to the trion. Eventually the trion intensity becomes the dominant emission channel. This behavior is consistent with our hypothesized model of a transition to the 1T phase^15^. As discussed previously, doping could cause a similar reduction in PL intensity. However, as discussed above very little if any analyte remains on the film while the PL spectra are taken. Because the film retains its lower intensity PL state after vacuum annealing at temperature below the likely transition temperature (~400 °C), where given the binding energies of adsorbates it is extremely unlikely that they persist, doping is not a probable cause of our observed behavior. The persistence of the trion peak indicates excess charge remains in the film^7^. In a mixed phase material, the areas of the film that are 1T could be a source of this excess charge. The formation and dissolution of trions during the charge transfer process has been theoretically shown to result in changes to the sample mobility^7^. This behavior is therefore also consistent with the increased conductance observed during the active measurements reported above.

Additionally, oxidation can be ruled out as a cause of the PL intensity decrease (and, consequently, as a cause of all of the exposure data presented here). As can be seen in Fig. 4, the sample intensity recovered after annealing at 400 °C in vacuum for 2 hours, which is unlikely to be sufficient for thermal reduction. Moreover, as DFT calculations and other research confirms, oxygen species are charge acceptors, which would result in the suppression of the trion peak for our *n*-type samples^5, 6^, opposite of what we observed^7, 31, 32, 38^.

In Fig. 4g the peak position of the neutral exciton (blue circles) and trion (red diamonds) is plotted as a function of TPA exposure. The peak separation of ~20 meV is on the order of the trion binding energy reported elsewhere for MoSe_2_^41^. In addition to becoming the dominant emission channel, we observed a peak shift in the trion as a function of exposure. A peak shift during a phase transition was observed previously in MoS_2_, and may be attributable to strain^10, 41, 42^. While this seems to be a reasonable explanation given the intimate relationship between strain and the phase change^10^, we did not observe a significant change in the Raman peaks as might be expected for strained materials (see Supplemental Information)^42^. It is more likely that the shift in trion position is due to band gap renormalization associated with the increase in charge density in the film^43, 44^.

**Figure 5:**
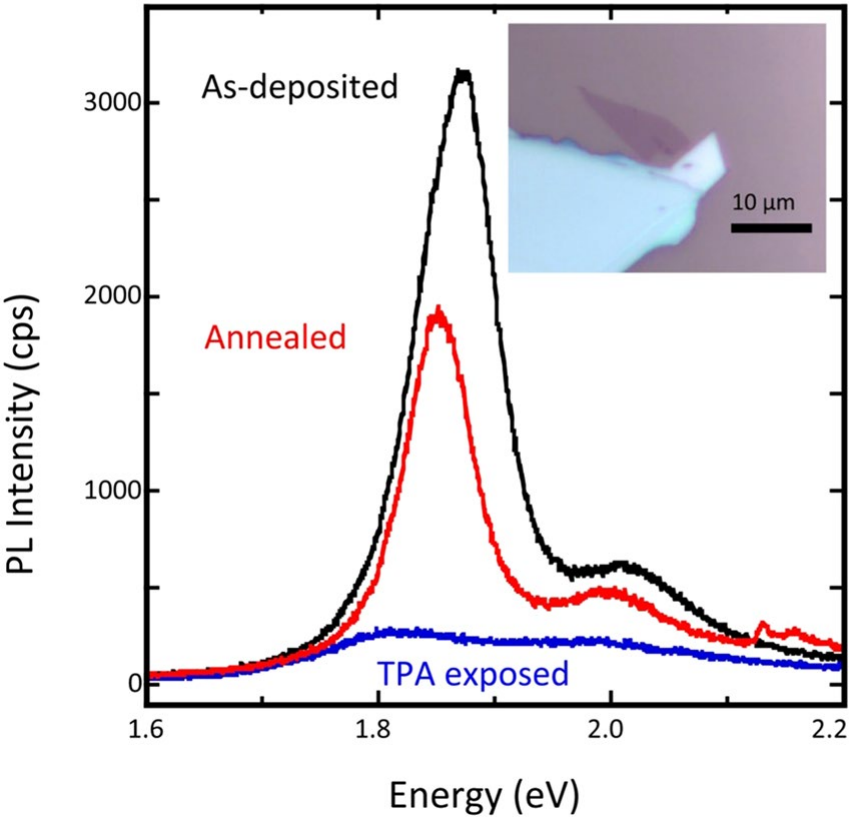
PL spectra of a MoS_2_ film for as-deposited (black) and after 10 days of exposure to TPA (blue) and XPS performed in UHV. There was almost a full extinction in peak intensity and a ~10 meV redshift in energy, indicating a partial 2H-1T phase change. The red peak corresponds to the PL after annealing for 2 hrs. in vacuum at 450 °C.

While the behavior we present in Fig. 4 is for a specific MoSe_2_ film, a similar pattern was seen in other samples as well. Figure 5 shows PL spectra taken on a MoS_2_ film exposed to TPA for 10 days. The black line shows the as-deposited emission, while the blue line shows the emission after 10 days of exposure and after acquiring XPS data in UHV (discussed below). We observe an almost complete quenching of emission. The red line shows the recovery of the emission after annealing in vacuum for 2 hrs. at 450 °C. The MoS_2_ film qualitatively behaves similarly in all respects to the MoSe_2_ film. Based on the predicted lower phase transition energy of MoSe_2_ vs. MoS_2_^10^, we would expect the transition to occur with less exposure for MoSe_2_, which does appear to be the case, at least qualitatively. It is interesting to note that the actively measured devices transitioned faster (we roughly estimate 5 × 10^3^ times faster) than the passively measured films. It was shown theoretically that an electric field, such as applied by a back gate, could aid in the charge transfer process, which would cause a faster phase transition^32^. Our data appear to support this theoretical result.

As a final additional method of corroboration, we performed XPS analysis of an exposed MoS_2_ film. We found the measurement to be largely inconclusive, with little change in the XPS spectrum before and after exposure even though the sample maintained a suppressed PL intensity even after the XPS performed in UHV. Detailed information and discussion can be found in the Supplemental Information.

## Conclusion

When taken together, the transport, Raman, and PL data provide strong evidence that the MoX_2_ films have substantially transitioned from the 2H phase to the 1T phase due solely to chemical vapor exposure. The hallmarks of this transition in our sensor devices is the change from a low conductance, actively sensing state to a high conductance, unresponsive state following extensive analyte exposure. The PL peak quenching and subsequent recovery in both MoX_2_ films and the appearance of the 1T-phase MoS_2_ J1, J2, and J3 Raman peaks further supports the conclusion of a phase change. It is unclear at this time whether the transition that we report is localized to small islands in the film or includes domains across the majority of the film, although transport data supports the small islands hypothesis. We have extensively considered doping by adsorbed analytes and oxidation as causes of the observed conductance and optical data, as under certain circumstances, doped films would respond similarly. But, we can eliminate doping as the cause of our observed behavior because, in summary: (1) the conductance change is abrupt, terminal, and stable, with a complete cessation of device response to additional pulsed analyte exposure; (2) Additional transport data, such as FET measurements, do not follow the behavior expected for the doping model; (3) The likely transitioned device is stable under vacuum, for days on a shelf, and after annealing below a likely transition temperature—all conditions for which surface dopants are extremely unlikely to persist on the film, substantiated by previous studies and the desorption that we observe in the fast and slow recovery components of the conductance response data, and; (4) The optical data are entirely consistent with a mixed phase film as seen in previous studies in the literature, and the stability of the optical properties under the conditions cited above again effectively rule out persistent dopants as a cause of the behavior.

If the phase transition could be harnessed to directly sense strong electron donor analytes it would create an entirely new chemical vapor sensing paradigm, whereby passive-type optical measurements could be combined with or used separately from active conductance measurements for the identification of analyte vapors all with the same device. As most chemical vapor analytes of interest (for instance, nerve gas and explosive by-products and constituents) are strong electron donors or acceptors, we propose that the 2H-1T phase transition can be used as the operating mechanism for a new method of identifying chemical compounds. The presence of a strong electron donor will cause a phase change in the 2H state, thus signaling the presence of a possibly dangerous vapor. As the amount of charge necessary to induce a phase change in each TMD material is different, a suite of concurrently sensing TMD materials could allow various strength electron donors/acceptors to be sensed and even identified with the necessary redundancy to minimize error^45^. Furthermore, the phase transition should be infinitely cycle-able with annealing, and TMDs are inherently flexible and relatively inexpensive to produce.

## Electronic supplementary material

Supplemental Information
