# Supplementary material for: Evidence for Chemical Vapor Induced 2H to 1T Phase Transition in MoX2 (X = Se, S) Transition Metal Dichalcogenide Films
Source: Sci Rep. 2017 Jun 19;7:3836. doi: 10.1038/s41598-017-04224-4 (PMC5476674; doi:10.1038/s41598-017-04224-4)
Supplement: Supplementary file 1 — Supplemental Information [file 41598_2017_4224_MOESM1_ESM.doc]

**Supplemental Information for Evidence for Chemical Vapor Induced 2H to 1T Phase Transition in MoX_2_ (X=Se, S) Transition Metal Dichalcogenide Films**

Adam L. Friedman^1*^, Aubrey T. Hanbicki^1^, F. Keith Perkins^2^, Glenn G. Jernigan^2^, James C. Culbertson^2^, and Paul M. Campbell^2^

*^1^Materials Science and Technology Division, Naval Research Laboratory, 4555 Overlook Ave., S.W., Washington, DC, USA 20375*

*^2^Electronics Science and Technology Division, Naval Research Laboratory, 4555 Overlook Ave., S.W., Washington, DC, USA 20375*

*Address correspondence to: adam.friedman@nrl.navy.mil

**Additional Electrical Data**

In **Fig. S1(a)**, we present IV curves for an as-fabricated MoSe_2_ device, the same device after a saturation dose of TEA, and after a 2-hour vacuum anneal at 400 °C. We have used a semi-log scale to make comparison easier. It is important to note that large double Schottky barriers necessitate using a higher bias voltage to reach the linear portion of the curve. The resistance of the as-fabricated device is ~25 GΩ, while the dosed device (partially transitioned) has a resistance of ~228 MΩ, a change of approximately 2 orders of magnitude.

In **Fig. S1(b)**, we present a supplemental figure to Fig. 2(d) in the main text: current vs. gate voltage for as fabricated, TEA exposed, and vacuum annealed device. This is the same device as presented in Fig. 2(d), but we present here a wider gate voltage range. The exposed, partially transitioned device turns on much faster and has conductance behavior comparable to other semiconductor channel devices with metallic inclusions, as discussed in the main text.

**Figure S1: (a) Semi-log plot of current vs. voltage for an as-fabricated, saturation- dosed, and vacuum annealed MoSe_2_ device. (b) Current vs. gate voltage for the device presented in Fig. 2(d) in the main text with a wider range than was presented there.**

**Active Sensing with MoS_2_**

We performed active electrical measurements on MoS_2_ FET devices using the home-built chemical vapor sensing apparatus and methods described in the main text. **Figure S2** summarizes these experiments, which resulted in the observation of behavior similar to the MoSe_2_ FET devices described in the main text. All devices responded to TEA, as discussed elsewhere.[[1]](#endnote-2), [[2]](#endnote-3) After a series of pulse sequences, the device response degrades and eventually stops. Initial resistance at a back gate voltage of 10 V was ~1 MΩ. The resistance decreased to ~18 kΩ after a number of TEA pulsed sequence exposures. Subsequent pulse sequences show unresponsive devices that must be annealed to recover functionality.

**Figure S2: Active sensing measurements with MoS_2_ FET device. The red line shows a response to a series of TEA pulses. After several pulse sequences (not of the same cycle times), the device response degrades and stops, shown in blue. Finally, the device no longer responds to analyte (black) and must be annealed to recover its responsiveness.**

**Additional Raman Spectroscopy Analysis**

**Fig. S3** shows a wider range plot than in the main paper showing the Raman scan from the point indicated on the MoSe_2_ flake image by the black dot for a variety of exposures times and annealing temperatures. Raman maps of the entire film were acquired. The images on the right in **Fig. S3** show the integrated intensity of the A_1g_ and E^1^_2g_ peaks over the entirety of the film. The intensity plots are superimposed over optical images. The Raman spectra were quite uniform over this area.


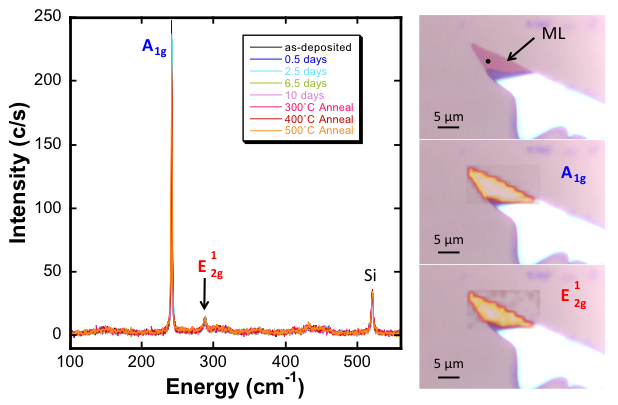


**Figure S3: Wide range Raman plot showing the two main peaks for MoSe_2_. The black dot on the right upper optical image indicates the location from which the data were taken. The middle and lower right images show a superimposition of the integrated intensities of the A_1g_ and E^1^_2g_ and the optical images, attesting to the uniformity of the sample.**

**Fig. S4** (left plot) shows the same data as **Fig. S3** plotted on a log scale. As can be seen, there are no additional peaks to be found that are greater than the noise. The panels on the right plot the peak positions of the A_1g_ and E^1^_2g_ peaks as a function of exposure or annealing event. Previous studies have shown that strain is associated with a significant amount of Raman peak mobility.[[3]](#endnote-4) We observe no significant peak mobility in our data.


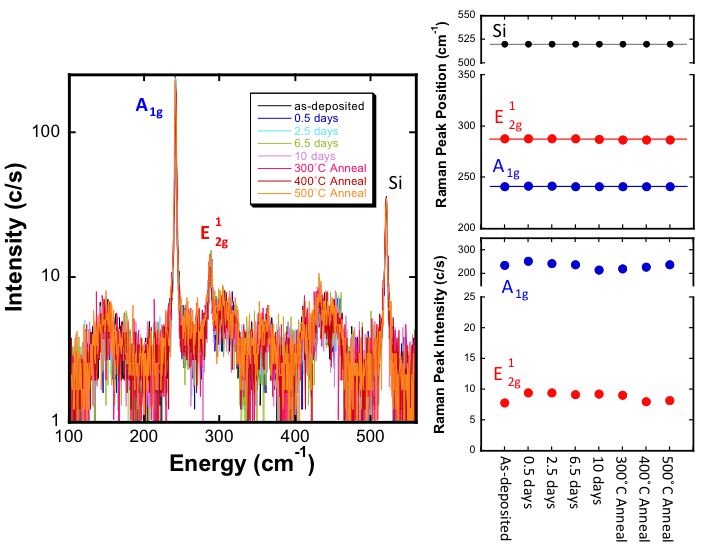


**Figure S4: The left image is the same Raman data as plotted in Fig. S2 on a log-plot showing that there are no additional peaks in the spectra arising out of the noise. On the right, the peak positions as a function of exposure or annealing event for both the A_1g_ and E^1^_2g_ peaks. The peaks do not move very much.**

**XPS Studies**

As an additional method of corroboration, we performed XPS analysis of a MoS_2_, which we found to be largely inconclusive. As discussed in the main text, both doping and a phase transition can cause a ~1 eV shift in binding energy for the Mo peaks.[[4]](#endnote-5) We were unable to measure the S peaks due to their proximity to the Si peak from our substrate. But, as discussed in the main text, measurements of shifts in S peaks are also largely un-attributable to one effect or another, as they can result from both a phase change and from vacancies. As seen in **Fig. S5**, the before and after vapor treated film showed no appreciable shift in binding energy. This is in spite of PL scans that show that after performing both the chemical vapor treatment and the XPS scan (in UHV), the sample retained its lower intensity behavior (see **Fig. 5,** main text). Our XPS collection area is ~50,000 μm^2^, much larger than the ~40 μm^2^ of our monolayer sample. So, in addition to the localized monolayer that we were attempting to probe, we also sampled many very thick stacks of material near the monolayer. This can be seen in the optical image of the sample in **Fig. S5**. As the vapor exposure process only exposes the top layer of material, and X-rays penetrate through the entire stack of material, it is difficult to discern any phase transitioned/doped areas from unaffected areas around them. In the best-case scenario, we would expect to see a broadening in the XPS after chemical vapor exposure, indicating the ~1 eV shifted top layer contribution. However, as the monolayer constitutes a small fraction of the total signal, we would see a proportional amount of broadening. We perhaps see a slight broadening in **Fig. S5.** However, in general, the XPS before and after chemical vapor exposure is unchanged, resulting in an inconclusive measurement.


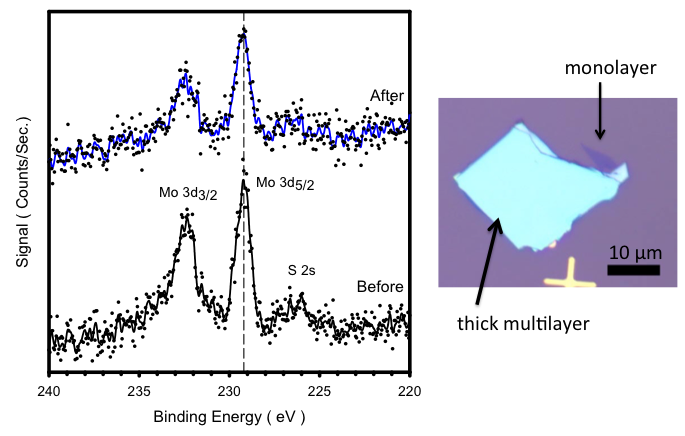


**Fig. S5: XPS spectrum of an as deposited sample (black, before), and a sample treated in TPA for 10 days (blue, after). The optical image on the right identifies the monolayer flake and a large, thick multilayer stack next to it that is likely obscuring our results. There were multiple similar thick stacks in the XPS collection area.**

**References:**

1. Perkins, F.K.; Friedman, A.L.; Cobas, E.; Campbell, P.M.; Jernigan, G.G; Jonker, B.T. Chemical vapor sensing with monolayer MoS_2_. *Nano Lett*. **2013**, 13, 668-673. [↑](#endnote-ref-2)
2. Friedman, A.L.; Perkins, F.K.; Cobas, E.; Jernigan, G.G.; Campbell, P.M.; Hanbicki, A.T.; Jonker, B.T. Chemical vapor sensing to two-dimensional MoS_2_ field effect transistor devices. *Sol. St. Elec*. **2014**, 101, 2-7. [↑](#endnote-ref-3)
3. Rice, C.; Young, R.J.; Zan, R.; Bangert, U.; Wolverson, D.; Gerogiou, T.; Jalil, R.; Novoselov. Raman scattering measurements and first-principles calculations of strain-induced phonon shifts in monolayer MoS_2_. *Phys. Rev. B*, **2013**, 87, 081301(R). [↑](#endnote-ref-4)
4. Wi, S.; Kim, H. Chen, M.; Nam, H.; Guo, L.J.; Meyhofer, E.; Liang, X. Enhancement of photovoltaic response in multilayer MoS2 induced by plasma doping. *ACS Nano* **2014**, 8, 5270. [↑](#endnote-ref-5)
